# Liu-pao Tea as a Source of Botanical Oviposition Attractants for *Aedes* Mosquitoes

**DOI:** 10.3390/insects16101065

**Published:** 2025-10-17

**Authors:** Li-Hua Xie, Tong Liu, Wen-Qiang Yang, Yu-Gu Xie, Si-Yu Zhao, Xiao-Guang Chen

**Affiliations:** 1 Department of Pathogen Biology, Institute of Tropical Medicine, School of Public Health, Southern Medical University, Guangzhou 510515, China; 2 Department of Pathogen Biology, School of Basic Medical Sciences, Fujian Medical University, Fuzhou 350122, China

**Keywords:** *Aedes*-borne diseases, volatile organic compounds, botanical infusion, oviposition attractants, GC-MS

## Abstract

Oviposition attractants can enhance both the surveillance and control of container-breeding *Aedes* mosquitoes. Although green tea has been reported to attract *Ae. aegypti*, the specific bioactive botanical compounds responsible remain unidentified. We evaluated teas across fermentation stages (non-fermented, fully fermented, and post-fermented) at 5 g/L for gravid *Ae. albopictus* using dual-choice assays. The most attractive tea (Liu-pao) was further tested across concentrations (0.5–5 g/L) and infusion ages (1–28 days). Autoclaved vs. non-autoclaved infusions assessed microbial contributions, while headspace volatiles were analyzed via HS-SPME-GC-MS. Individual compounds (cedrol and linalool oxide) were bioassayed. Only Liu-pao tea consistently attracted *Ae. albopictus* (OAI ≥ 0.3), peaking at 5 g/L after 7–14 days (OAIs 0.73/0.67). A 0.5 g/L infusion aged 21–28 days also attracted *Ae. aegypti* (OAIs 0.89/0.63). Autoclaving did not reduce attraction, confirming volatile compounds, not live microbe-mediated effects. Cedrol (1 mg/L) elicited moderate attraction (OAI 0.29) in *Ae. albopictus*, while linalool oxide was inactive. Post-fermented Liu-pao tea acts as a potent botanical attractant for *Aedes* mosquitoes. Cedrol contributes partially, but whole tea infusions outperform single compounds. These findings support developing multi-volatile “attract-and-kill” strategies targeting gravid mosquitoes.

## Simple Summary

Diseases transmitted by *Aedes* mosquitoes, including dengue, Zika, and chikungunya, lack effective vaccines and specific treatments, making vector control critical for disease prevention. Oviposition attractants offer a promising tool for managing mosquito populations. Effective oviposition attractants for *Aedes* mosquitoes are urgently needed. While green tea attracts *Aedes aegypti*, it is unclear how different tea fermentations affect the Asian tiger mosquito (*Aedes albopictus*). We tested teas across fermentation stages, evaluating attraction, the role of microbes, and chemicals. Results showed aged Liu-pao tea strongly attracts them at 5 g/L after 1–2 weeks. Notably, even a weak Liu-pao (0.5 g/L) solution successfully attracted *Ae. aegypti* mosquitoes with an oviposition activity index (OAI) of 0.89 (day 21). Crucially, we discovered that this attraction was not due to living microbes in the tea water, but rather natural chemicals released from the tea leaves. Also, we identified one specific chemical, cedrol, as part of this attraction, but found that the full tea solution worked much better than cedrol alone. These findings establish Liu-pao tea as a potent botanical attractant and support the development of multi-volatile “attract-and-kill” strategies for gravid *Aedes* mosquitoes.

## 1. Introduction

Gravid mosquitoes rely on chemosensory cues (olfactory, gustatory, or both) to identify suitable oviposition sites. In nature, these cues are complex, typically involving plant infusions, microbial activity, conspecific immatures, and even predator presence. Preferences vary among mosquito genera such as *Anopheles*, *Culex*, and *Aedes* [1,2,3]. Because larvae and pupae are restricted to aquatic habitats, oviposition site selection is a crucial determinant of offspring survival. Characteristics of favorable sites include nutrient availability, absence of predators, and reduced competition [4].

In recent years, multiple studies have shown that bacterial metabolites in breeding-site water strongly influence oviposition choice [5,6]. For example, Ponnusamy et al. analyzed volatiles from white oak leaf infusions via GC-MS and identified a blend of nonanoic acid (16%), myristic acid (83%), and methyl myristate (1%) that stimulated *Aedes aegypti* (*Ae. aegypti*) oviposition [7,8]. Similarly, Eneh et al. highlighted the importance of microbial flora in combination with physical and chemical factors in shaping oviposition behavior. Microbial isolates from plant infusions, such as *Bacillus subtilis* from oak leaves, were shown to stimulate oviposition [8,9,10].

Previous work demonstrates that *Aedes albopictus* (*Ae. albopictus*) frequently oviposits in small containers with plant detritus, though plant species preferences vary geographically. Importantly, *Aedes* mosquitoes exhibit “skip oviposition” behavior, often distributing eggs across multiple sites and showing a preference for artificial containers [4]. These cryptic breeding habitats complicate control efforts but also present opportunities to exploit oviposition attractants in surveillance and control strategies. Given the limited availability of effective oviposition attractants for *Aedes* mosquitoes, exploring plant-derived infusions with distinct fermentation processes represents a promising strategy. Green tea has been implicated in stimulating oviposition in *Ae. aegypti* [11], but the diversity of teas, especially their fermentation stage, quality [12,13], and microbial communities [8] may differentially affect attractiveness. The role of fermentation processes in modulating attractiveness remains unexplored. Post-fermented Liu-pao tea undergoes extensive microbial transformation during manufacturing, producing volatile organic compounds distinct from non-fermented or oxidized tea varieties, which may replicate the microbial signatures associated with productive mosquito breeding habitats. Given its regional availability in arbovirus-endemic areas of southern China, Liu-pao tea represents a promising botanical source for developing sustainable, field-deployable oviposition attractants [14,15,16].

Moreover, the role of their volatile constituents in influencing *Ae. albopictus* behavior remains unclear. To address this gap, we systematically compared teas at different fermentation stages and evaluated their oviposition activity in dual-choice assays. In addition, microbial inactivation and chemical profiling were performed to disentangle the relative contributions of microbial activity and plant-derived volatiles. We hypothesized that post-fermented Liu-pao tea would exhibit stronger and more consistent oviposition stimulation than non- or fully fermented teas, and that specific volatile organic compounds would mediate this effect. By identifying and validating bioactive compounds, this study aims to advance the development of natural oviposition attractants and provide a basis for botanical “attract-and-kill” strategies targeting container-breeding *Aedes* mosquitoes.

## 2. Materials and Methods

### 2.1. Mosquitoes

Laboratory-reared strains of *Ae. albopictus* were field-collected in Foshan, while *Ae. aegypti* were sourced from Haikou, Hainan, provided by the Guangdong CDC. Colonies were maintained under standardized laboratory conditions (27 °C ± 1 °C; 75% ± 5% relative humidity; 14:10 h light–dark cycle) to ensure physiological consistency. Larvae were reared on fish food, and adults sustained with 10% sucrose solution.

### 2.2. Main Compounds

Compounds used included cedrol (≥98%, Aladdin, Shanghai, China) and linalool oxide (≥97%, Aladdin, Shanghai, China). Three teas representing distinct fermentation levels were selected: non-fermented Jiangxi green tea (Ningdu Xiaobuyan Tea Industry Co., Ltd., Ganzhou, China), fully fermented Tianlao black tea (Xinchang Caiyan Xiandao Tea Co., Ltd., Xinchang, China), and post-fermented Guangxi Liu-pao tea (Wuzhou Tea Factory Co., Ltd., Wuzhou, China).

### 2.3. Dual-Choice Oviposition Assays

**Figure 1:**
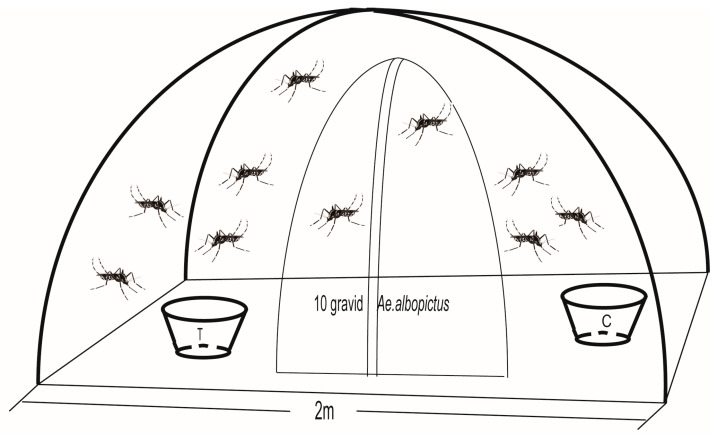
Experimental set up for dual-choice oviposition assays.

Ten gravid females were released into a 2.0 × 1.8 m Mongolian yurt net enclosure (Figure 1). In all oviposition preference bioassays, tea was steeped in overnight-settled tap water (1000 mL) to eliminate residual chlorine. Meanwhile, the control group consisted of overnight-settled tap water (1000 mL), which was kept at room temperature (25 °C ± 2 °C). Each experimental container and control container were placed equidistantly within the Mongolian yurt net to minimize positional bias. Additionally, the positions were randomized and rotated between replicates. After 48 h, eggs were counted to calculate:

oviposition rate= Nt / (Nt + Nc )

OAI = (Nt−Nc ) /(Nt + Nc )

where Nt = eggs in treatment and Nc = eggs in control. OAI ranges from −1 (avoidance) to +1 (exclusive preference) with values > 0.3 were biologically significant [17]. Each group of experiments consisted of 6 replicates. The number of females that successfully oviposited (fully or partially) was recorded. Groups with an average egg production per female mosquito > 30 were included, while those with an average egg production per female mosquito < 10 were considered non-gravid or physiologically unable to oviposit and were excluded from analysis.

Mongolian yurt enclosure (2.0 × 1.8 m) with two oviposition cups (treatment vs. control) positioned symmetrically. Ten gravid *Aedes* mosquitoes were released for 48 h to evaluate oviposition preference.

### 2.4. Screening Tea Infusions

To establish baseline oviposition behavior and assess potential positional bias, we conducted control vs. control experiments (n = 6 replicates) where gravid females were offered two identical containers with tap water only. Tea infusions were prepared at 5 g/L (based on prior evidence that 16.8 g/L plant infusions attract *Aedes* [6]). Infusions were refreshed every 3 days to maintain chemical stability. Liu-pao tea (post-fermented) was additionally evaluated at 0.5, 1.5, and 5 g/L, with weekly refreshments to assess dose-dependent effects. All concentrations were tested over 0–28 days.

### 2.5. Investigation of Key Liu-pao Tea Components

#### 2.5.1. Role of Microbes in Oviposition Attraction

To determine whether the attractiveness of Liu-pao tea is due to compounds inherently present in the tea leaves or those produced by microbial activity during fermentation and storage, we compared autoclaved vs. non-autoclaved tea infusions. Both autoclaved and non-autoclaved tea samples (5 g/L) were prepared by soaking in overnight-settled tap water for 24 h at 25 °C. Autoclaved samples were sterilized (121 °C, 20 min) and immediately used for oviposition experiments, while non-autoclaved samples followed the same preparation procedure but without sterilization. To isolate microbial contributions at different doses and time points in Liu-pao tea, autoclaved (sterile) vs. non-autoclaved Liu-pao tea infusions, non-autoclaved Liu-pao tea infusions vs. blank control and autoclaved Liu-pao tea infusions vs. blank control were tested to attract *Ae. albopictus* to lay eggs at 0.5–5 g/L over 7–28 days, respectively. OAIs were calculated from 3 to 6 replicates per treatment.

#### 2.5.2. Volatile Identification via GC-MS

Infusions with divergent oviposition effects (0.5 g/L at 1 day; 1.5 g/L at 14 days; 5 g/L at 14 days) were analyzed. Volatiles were extracted from the headspace using HS-SPME (Supelco, Bellefonte, PA, USA) and identified via GC–MS (analysis performed by Biotree Technologies, Shanghai, China) (detailed parameters in Table S1, Supplementary Materials). Briefly, Agilent 7890B GC (Agilent Technologies, Santa Clara, CA, USA) was coupled with 5977A MSD; DB-5MS column (Agilent Technologies, CA, USA) (30 m × 0.25 mm × 0.25 μm); temperature program 40 °C (4 min) to 245 °C at 5 °C/min; EI mode 70 eV; scan range *m*/*z* 20–500. Compounds were identified by comparing mass spectra with NIST17 library (match quality ≥ 85%) and confirmed using retention indices and authentic standards where available. Cedrol (0.01–10 mg/L) and linalool oxide (0.00001–0.01 mg/L) were bioassayed for standalone activity.

#### 2.5.3. Data Processing

GC–MS data were processed using ChromaTOF (LECO Corporation, USA, v4.3x) and the NIST library. Standard pipelines included peak extraction, baseline correction, spectrum convolution, peak integration, deconvolution, and alignment [14].

### 2.6. Statistical Analysis

Data were analyzed in SPSS 20.0 (IBM, Armonk, NY, USA). Groups were compared via Student’s *t*-test. Oviposition attraction was defined as oviposition rate > 50% and OAI > 0, with OAI > 0.3 indicating significant attraction [11]. Graphs were generated in GraphPad Prism version 8.0 (San Diego, CA, USA).

## 3. Results

### 3.1. Tea Infusions Screening for Ae. albopictus Oviposition

**Figure 2:**
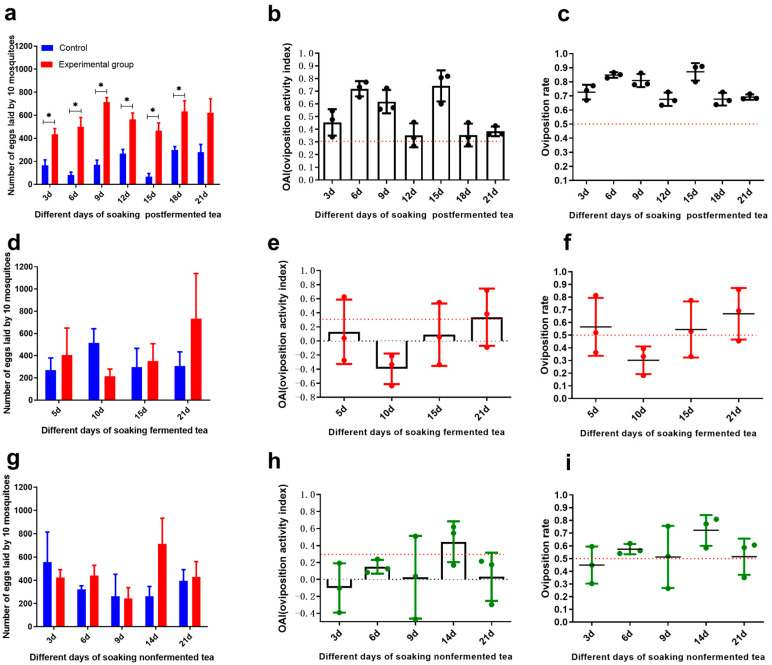
Oviposition response of *Ae. albopictus* to three tea infusions (5 g/L) over 28 days. (**a**) Post-fermented tea: Mean eggs per 10 mosquitoes (±SD), (**b**) OAI (±SE), and (**c**) oviposition rate (±SE). (**d**) Fermented tea: Mean eggs per 10 mosquitoes (±SD), (**e**) OAI (±SE), and (**f**) oviposition rate (±SE). (**g**) Non-fermented tea: Mean eggs per 10 mosquitoes (±SD), (**h**) OAI (±SE), and (**i**) oviposition rate (±SE). Significance: * *p* < 0.05 (n = 3–6 replicates).

Results showed no significant difference in egg distribution between the two control containers (*t* = 0.90, *p* = 0.41), with eggs distributed approximately equally (OAI = −0.01 ± 0.01), confirming the absence of positional bias. A preliminary oviposition assay was conducted using teas with different degrees of fermentation (non-fermented, fully fermented, and post-fermented) at a concentration of 5 g/L, with infusion times assessed approximately every three days. The total egg counts observed (ranging from 460 to 1200 eggs per replicate with 10 females) were consistent with natural oviposition behavior where complete egg depletion rarely occurs in a single gonotrophic cycle. The observed egg counts thus reflect genuine oviposition preferences rather than differential retention rates. The results showed that post-fermented tea infusions significantly promoted oviposition at 3 d (*t* = 6.87, *p* = 0.002), 6 d (*t* = 8.43, *p* = 0.001), 9 d (*t* = 16.77, *p* < 0.0001), 12 d (*t* = 9.64, *p* < 0.0001), and 18 d (*t* = 5.95, *p* = 0.004) compared with the control. The average oviposition activity indices (OAIs) at 3, 6, 9, 12, and 18 days were 0.45, 0.72, 0.62, 0.35, and 0.38, respectively, all above the threshold OAI value of 0.3 (Figure 2a–c). By contrast, the other two tea types showed no significant differences from the control (Figure 2d–i). These findings suggest that post-fermented tea infusion is a promising botanical attractant for *Ae. albopictus* oviposition.

### 3.2. Dose-Response and Infusion Time Effects of Liu-pao Tea

**Figure 3:**
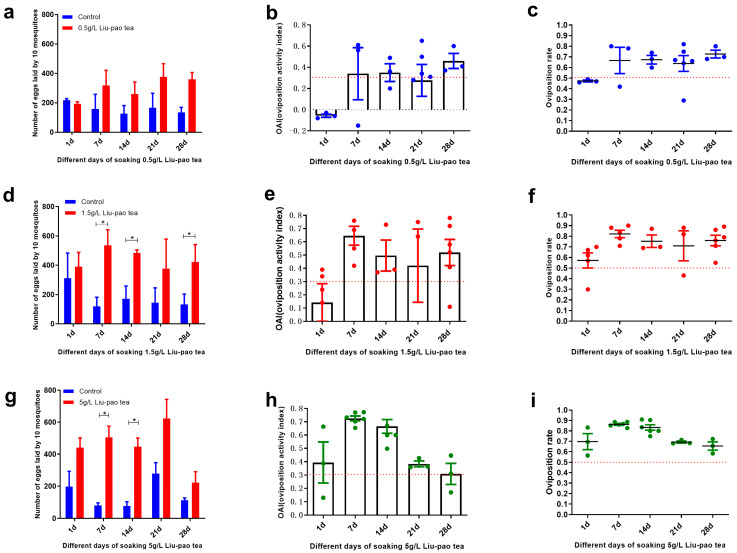
Dose-response and infusion time effects of Liu-pao tea infusions (0.5–5 g/L) on *Ae. albopictus* oviposition over 28 days. (**a**) 0.5 g/L Liu-pao tea: Mean eggs per 10 mosquitoes (±SD), (**b**) OAI (±SE), and (**c**) oviposition rate (±SE). (**d**) 1.5 g/L Liu-pao tea: Mean eggs per 10 mosquitoes (±SD), (**e**) OAI (±SE), and (**f**) oviposition rate (±SE). (**g**) 5 g/L Liu-pao tea: Mean eggs per 10 mosquitoes (±SD), (**h**) OAI (±SE), and (**i**) oviposition rate (±SE). Significance: * *p* < 0.05 (n = 6 replicates).

To further refine the evaluation of Liu-pao tea, infusions were tested at concentrations of 5 g/L, 1.5 g/L, and 0.5 g/L, and refreshed every seven days. Significant oviposition stimulation was observed for 5 g/Lat 7 d (*t* = 14.37, *p* < 0.0001) and 14 d (*t* = 13.65, *p* < 0.0001) (Figure 3g–i), and for 1.5 g/L at 7 d (*t* = 7.48, *p* < 0.0001), 14 d (*t* = 6.05, *p* = 0.004), and 28 d (*t* = 5.15, *p* = 0.0004) (Figure 3d–f). With the exception of 0.5 g/L (Figure 3a–c) and 1.5 g/L infusions at day 1 and day 21, all other treatments yielded mean OAI values above 0.3. Notably, 5 g/L infusions at 7 and 14 days exhibited particularly high OAIs of 0.73 and 0.67, respectively, exceeding the corresponding values of lower concentrations. Overall, higher concentrations of Liu-pao tea (up to 5 g/L) and infusion times of 7–14 days produced the strongest oviposition stimulations.

### 3.3. Cross-Species Attraction to Ae. aegypti

**Figure 4:**
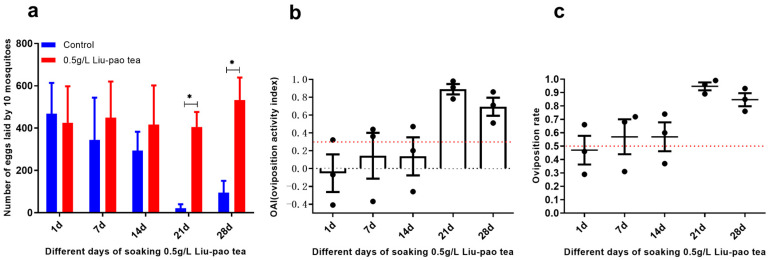
Attractiveness of infusion time of Liu-pao tea (0.5 g/L) to *Ae. aegypti* over 28 days. (**a**) Mean eggs per 10 mosquitoes (±SD), (**b**) OAI (±SE), and (**c**) oviposition rate (±SE). Significance: * *p* < 0.05 (n = 3–6 replicates).

At 0.5 g/L, Liu-pao tea infusions also induced oviposition in *Ae. aegypti* when soaked for 21 and 28 days, with average OAIs of 0.89 (*t* = 9.10, *p* = 0.0008) and 0.63 (*t* = 6.33, *p* = 0.003), respectively. Compared with *Ae. albopictus*, the oviposition response of *Ae. aegypti* occurred later (Figure 4).

### 3.4. Microbial vs. Volatile Contributions

**Figure 5:**
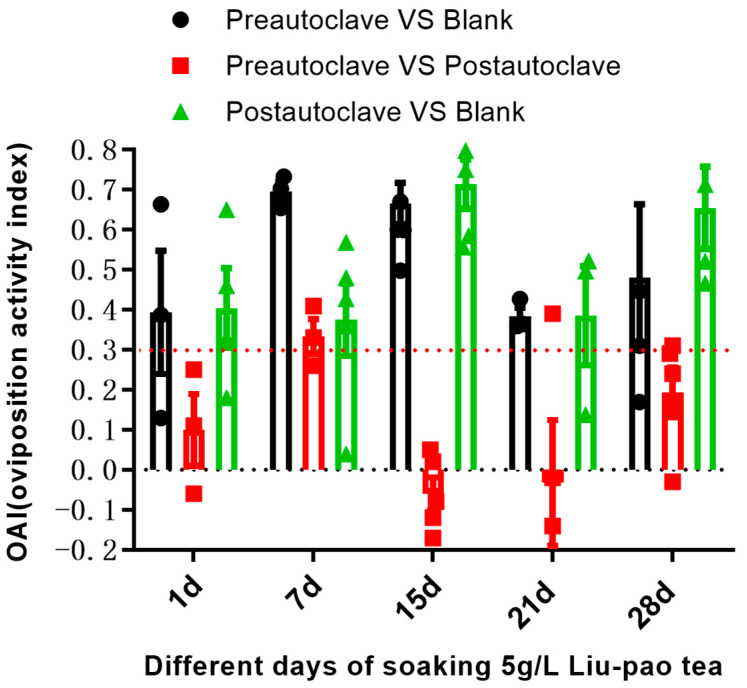
Comparison of oviposition response to autoclaved vs. non-autoclaved Liu-pao tea infusions. In black bars, Nt represents eggs laid in pre-autoclave, and Nc represents eggs laid in blank; in red bars, Nt represents eggs laid in pre-autoclave, and Nc represents eggs laid post-autoclave; in green bars, Nt represents eggs laid post-autoclave, and Nc represents eggs laid in blank. Data presented as mean ± SE (n = 3–6).

Autoclaving treatments were performed systematically to evaluate microbial contributions to oviposition attraction. At day 1, both 5 g/L pre-autoclave and post-autoclaved infusions compared to the untreated blank water group showed a moderate oviposition response (with OAI = 0.39 ± 0.15 and OAI = 0.41 ± 0.10), respectively. By day 7, the pre-autoclave infusions showed the strongest oviposition response (OAI = 0.70 ± 0.02), significantly outperforming both the post-autoclaved infusions (pre-autoclave infusions vs. post-autoclaved: OAI = 0.33 ± 0.04 and 5 g/L post-autoclaved vs. blank water: OAI = 0.38 ± 0.09). This indicates that the 5 g/L solution is the most effective treatment early in the experiment. At day 15 and day 21 the effectiveness of the pre-autoclave and post-autoclaved infusions were similar and pre-autoclave remained lower than the peak observed at day 7 (Figure 5). The similar attractiveness of autoclaved and non-autoclaved infusions demonstrates that the oviposition stimulants in Liu-pao tea are primarily heat-stable phytochemicals naturally present in or produced during the fermentation process of tea manufacturing, rather than compounds generated by bacterial activity during the short-term infusion period. This distinguishes Liu-pao tea from hay infusions, where active microbial fermentation during preparation is essential for attractiveness. The post-fermentation processing of Liu-pao tea during manufacturing involves the microbial transformation of tea compounds, but once these compounds are formed, they remain stable and bioactive even after sterilization.

### 3.5. GC-MS Identification of Oviposition-Active Volatiles

**Figure 6:**
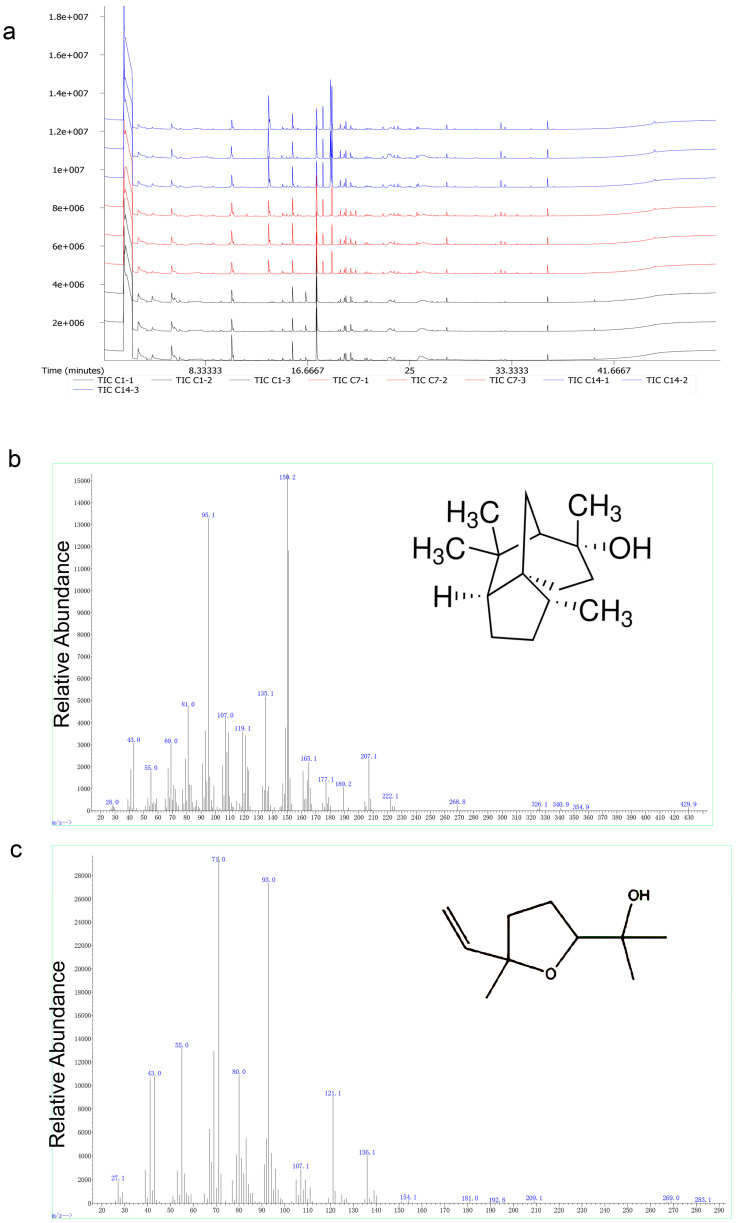
GC-MS chromatographic analysis of Liu-pao tea volatile compounds. (**a**) Total ion chromatogram (TIC) showing the complete volatile profile of Liu-pao tea infusion. The complex mixture contains numerous compounds with overlapping peaks. (**b**) Extracted ion chromatogram (EIC) specifically showing cedrol (retention time 32.771 min) and (**c**) linalool oxide (retention time 20.586 min). C1 (1–3) is 0.5 g/L soaked for 1 d, C7 (1–3) is 5 g/L soaked for 7 d, and C14 (1–3) is 5 g/L soaked for 14 d.

GC-MS analysis of Liu-pao tea extracts revealed 223 peaks in Figure 6a. Compounds with relative contents greater than 1% included seven in 0.5 g/L samples (1 day), eight in 5 g/L samples (7 days), and eight in 5 g/L samples (14 days). These compounds were classified into carbon–oxygen compounds, alkanes, ketones, benzenes, and nitrogen-containing species. Carbon dioxide, octamethylcyclotetrasiloxane, toluene, and nitrogen were consistently present across all time points. Importantly, the key aroma compounds linalool and cedrol, previously reported as major constituents of Liu-pao tea, were also identified (Figure 6b,c and Table 1).

### 3.6. Effects of Individual Aroma Compounds on Mosquito Oviposition

**Figure 7:**
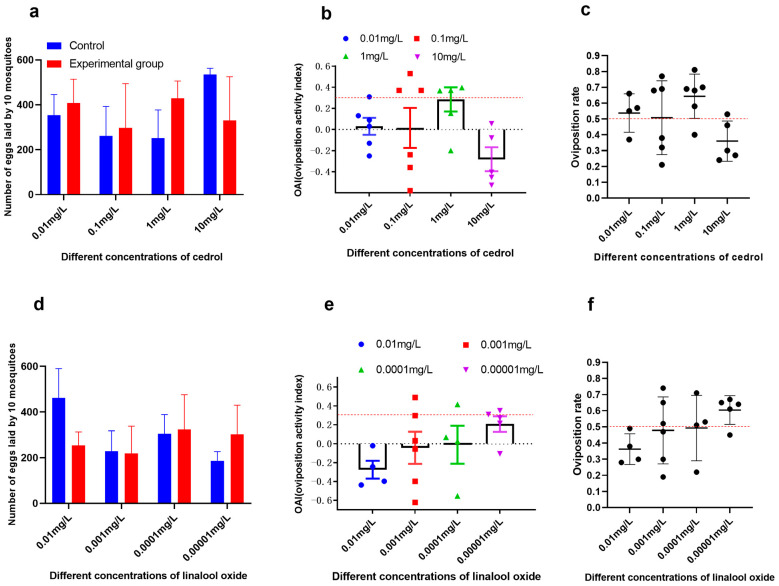
Bioactivity of identified volatiles against *Ae. albopictus* oviposition. (**a**) Cedrol: Mean eggs per 10 mosquitoes (±SD), (**b**) OAI (±SE), and (**c**) oviposition rate (±SE). (**d**) Linalool oxide: Mean eggs per 10 mosquitoes (±SD), (**e**) OAI (±SE), and (**f**) oviposition rate (±SE) (n = 3–6 replicates).

Bioassays confirmed cedrol (1 mg/L) as a potent attractant for *Ae. albopictus*, at 1 mg/L, cedrol showed a trend toward attractiveness with an average oviposition rate of 64% (>50%) and an average OAI of 0.29, which is between 0 and 0.3. The absolute number of eggs laid in cedrol-treated cups (429 ± 76) was not significantly different from controls (251 ± 125; *t* = 2.39, *p* = 0.62) (Figure 7a–c). In contrast, linalool oxide (tested at 0.00001–0.01 mg/L) exhibited no significant oviposition activity (OAI < 0.3) (Figure 7d–f), despite being abundant in Liu-pao tea infusions.

## 4. Discussion

The ecology of mosquito oviposition is a complex interplay between environmental cues, microbial activity, and chemical signaling, with plant-derived organic matter playing a pivotal role in shaping larval habitat selection. Plant debris in aquatic environments not only serves as a critical nutritional source for developing larvae but also emits a rich array of volatile organic compounds (VOCs) that act as semiochemicals, guiding gravid females to identify suitable oviposition sites. Our findings contribute to the growing body of literature showing that botanical infusions can be potent sources of these cues. Previous work has demonstrated the efficacy of plant infusions such as white oak (*Quercus alba*), Bermuda grass (*Cynodon dactylon*), and bamboo in stimulating oviposition across various mosquito species [5,6,7,8,13]. By systematically screening teas with varying degrees of fermentation, our study specifically identified post-fermented Liu-pao tea as a superior botanical attractant for *Ae. albopictus*, outperforming both non-fermented and fully fermented teas.

The increasing attractiveness of Liu-pao tea over time is consistent with findings from other studies on plant infusions, in which microbial metabolism gradually modifies the chemical composition, leading to the release of novel volatile compounds [14]. It is widely recognized that microbes can either directly affect oviposition or promote the production of attractants. For instance, specific bacteria such as *Bacillus subtilis* in oak leaf extracts have been demonstrated to be potent oviposition stimulants [9,10]. Similarly, the fermentation of Liu-pao tea is propelled by a diverse microbial community, encompassing fungi and bacteria, which transform the chemical constituents of the tea [15]. In contrast to these previous findings, our results, where autoclaved and sterilized infusions often maintained or even enhanced their attractiveness, strongly indicate that the primary oviposition stimulants in Liu-pao tea are non-living chemical compounds. These may include both volatile organic compounds (VOCs) inherent in the fermented tea leaves and secondary metabolites generated during the initial fermentation stages, which are stable and remain present even in the absence of viable microbes. This differentiation is crucial as it redirects the focus from live microbes to persistent chemical cues, thereby simplifying the formulation of attractants.

Our GC-MS analysis effectively characterized the volatile composition of Liu-pao tea, identifying a complex blend of 223 compounds. Among them, cedrol emerged as a crucial candidate, which had been previously recognized as a principal aromatic component of Liu-pao tea [16]. The bioassay results verified its function as a bioactive oviposition stimulant for *Ae. albopictus* at a biologically significant concentration of 1 mg/L. This discovery is in line with extensive ecological understanding, as cedrol has been reported to trigger oviposition in other mosquito species such as *Anopheles gambiae* [6] and is a well-known volatile produced by soil fungi associated with grass roots in the vicinity of natural larval habitats [18]. Despite the high abundance of linalool oxide, it exhibited no bioactivity, highlighting that the efficacy of attractants is contingent upon specific VOC profiles. This further supports the hypothesis that mosquitoes employ a set of evolutionarily conserved plant- and microbe-derived VOCs as long-range and contact cues for habitat selection.

From a practical perspective, gravid mosquitoes have vulnerable life stages and are a promising target for control and surveillance strategies [19,20]. The discovery of a powerful, natural, and long-lasting attractant such as Liu-pao tea meets a crucial requirement in vector management. While cedrol showed marginal activity at low concentrations (1 mg/L), we did not observe a dose-dependent effect at higher concentrations (10 mg/L). This suggests that cedrol, along with other identified compounds, contributes significantly to the overall attractiveness of Liu-pao tea for *Aedes* oviposition. Its relatively weaker effect compared to the crude infusion suggests that the overall “attractant profile” of Liu-pao tea is likely synergistic, entailing a combination of volatile substances or their interaction with non-volatile components. This complexity highlights the benefit of utilizing the entire infusion, which serves as a stable, renewable, and cost-efficient lure. The identified compounds, especially cedrol, offer templates for the development of synthetic analogs.

Ultimately, these findings offer a robust scientific basis for the development of innovative “attract-and-kill” interventions. Such strategies entail the deployment of an attractant, such as Liu-pao tea infusion or a synthetic mixture emulating its volatile compounds, in conjunction with a lethal agent, for instance, a biological larvicide (*Bacillus thuringiensis subsp. israelensis*, *Bti*) or an insect-growth regulator. This approach not only effectively targets gravid female mosquitoes but also aggregates them in a specific location, thereby enhancing the efficiency of control measures and mitigating the overall environmental impact of broad-spectrum insecticides. This research lays the groundwork for field trials to verify the efficacy of Liu-pao tea-based traps for the surveillance and control of *Aedes* mosquitoes.

Although this study validates Liu-pao tea as a proof-of-concept botanical attractant, its efficacy requires field verification across diverse ecological contexts, particularly in relation to natural oviposition sites. The moderate activity of purified cedrol suggests the presence of uninvestigated synergistic interactions among multiple volatile compounds, and the post-fermentation mechanisms remain poorly understood. A detailed microbiome analysis during tea processing may uncover specific microbial metabolites responsible for the production of bioactive compounds. Future research priorities include large-scale field trials assessing Liu-pao tea-baited traps for both surveillance and “attract-and-kill” applications integrated with larvicides (e.g., *Bacillus thuringiensis israelensis*), the optimization of formulations for stability and application, comprehensive chemical profiling to identify synergistic effects, and cost–benefit analyses in comparison with synthetic alternatives. Filling these knowledge gaps will accelerate the translation of our findings into practical vector control programs.

## 5. Conclusions

The research findings indicate that Liu-pao tea encompasses multiple oviposition-active compounds, among which cedrol has been identified as a moderate bioactive volatile. Nevertheless, the relatively moderate activity of cedrol in isolation, when compared to the entire tea infusion, implies that synergistic interactions among multiple chemical constituents probably augment overall attractiveness. The specific combinations and interactions of these compounds necessitate further exploration. Moreover, field validation studies are requisite to ascertain whether Liu-pao tea-based attractants can compete effectively with natural oviposition sites under realistic circumstances.

## Figures and Tables

**Table 1:** Relative content of aroma substances in GC-MS samples.

Sample Number	Linalool Oxide (Linalool) %	Cedrol %
C 1-1	0.0194	0.0073
C 1-2	0.0212	0.0085
C 1-3	0.0336	0.0148
C 7-1	0.1465	0.0216
C 7-2	0.190	0.0335
C 7-3	0.3536	0.0389
C 14-1	0.0588	0.0279
C 14-2	0.0266	0.0133
C 14-3	0.0235	0.0135

NOTE: C1 is 0.5 g/L soaking for 1 d sample, wherein C7 is 5 g/L soaking for 7 d, and C14 is 5 g/L soaking for 14 d, l-3 corresponding three repeats.

## Data Availability

The datasets used and/or analyzed during the current study are available from the corresponding author on reasonable request.

## Supplementary Materials

The following supporting information can be downloaded at: https://www.mdpi.com/article/10.3390/insects16101065/s1, Table S1. Instrument parameters.

## Abbreviations

The following abbreviations are used in this manuscript:

OAI	oviposition activity index
HS-SPME-GC-MS	headspace solid-phase microextraction-mass spectrometry coupled with gas chromatography-mass spectrometry
VOCs	volatile organic compounds
*Ae. albopictus*	*Aedes albopictus*
*Ae. aegypti*	*Aedes aegypti*
*Bti*	*Bacillus thuringiensis* subsp. *israelensis*

## References

[1] Malassigné S., Laÿs M., Vallon L., Martin E., Meiffren G., Vigneron A., Van V.T., Minard G., Moro C.V., Luis P. (2025). Environmental yeasts differentially impact the development and oviposition behavior of the Asian tiger mosquito *Aedes albopictus*. Microbiome.

[2] Afify A., Galizia C.G. (2015). Chemosensory Cues for Mosquito Oviposition Site Selection. J. Med Entomol..

[3] Zhao S.Y., Wu P.L., Fu J.Y., Wu Y.M., Liu H.K., Cai L.J., Gu J.B., Zhou X.H., Chen X.G. (2024). Gustatory receptor 11 is involved in detecting the oviposition water of Asian tiger mosquito, *Aedes albopictus*. Parasit. Vectors.

[4] Chandel K., Suman D.S., Wang Y., Unlu I., Williges E., Williams G.M., Gaugler R. (2016). Targeting a Hidden Enemy: Pyriproxyfen Autodissemination Strategy for the Control of the Container Mosquito *Aedes albopictus* in Cryptic Habitats. PLOS Neglected Trop. Dis..

[5] Getachew E.B., Linus S., Richard T., Patrick O., Tullu B., Åsa E., Ulrike F. (2021). Grass-like plants release general volatile cues attractive for gravid *Anopheles gambiae sensu stricto* mosquitoes. Parasites Vectors.

[6] Eneh L.K., Saijo H., Borg-Karlson A.-K., Lindh J.M., Rajarao G.K. (2016). Cedrol, a malaria mosquito oviposition attractant is produced by fungi isolated from rhizomes of the grass Cyperus rotundus. Malar. J..

[7] Ponnusamy L., Xu N., Nojima S., Wesson D.M., Schal C., Apperson C.S. (2008). Identification of bacteria and bacteria-associated chemical cues that mediate oviposition site preferences by *Aedes aegypti*. Proc. Natl. Acad. Sci. USA.

[8] Ponnusamy L., Schal C., Wesson D.M., Arellano C., Apperson C.S. (2015). Oviposition responses of *Aedes* mosquitoes to bacterial isolates from attractive bamboo infusions. Parasites Vectors.

[9] Girard M., Martin E., Vallon L., Raquin V., Bellet C., Rozier Y., Desouhant E., Hay A.-E., Luis P., Moro C.V. (2021). Microorganisms Associated with Mosquito Oviposition Sites: Implications for Habitat Selection and Insect Life Histories. Microorganisms.

[10] Trexler J.D., Apperson C.S., Zurek L., Gemeno C., Schal C., Kaufman M., Walker E., Watson D.W., Wallace L. (2003). Role of Bacteria in Mediating the Oviposition Responses of *Aedes albopictus* (Diptera: Culicidae). J. Med Entomol..

[11] Snetselaar J., Andriessen R., A Suer R., Osinga A.J., Knols B.G., Farenhorst M. (2014). Development and evaluation of a novel contamination device that targets multiple life-stages of *Aedes aegypti*. Parasites Vectors.

[12] Li Y., Yu S., Yang S., Ni D., Jiang X., Zhang D., Zhou J., Li C., Yu Z. (2023). Study on taste quality formation and leaf conducting tissue changes in six types of tea during their manufacturing processes. Food Chem. X.

[13] Ponnusamy L., Xu N., Böröczky K., Wesson D.M., Ayyash L.A., Schal C., Apperson C.S. (2010). Oviposition Responses of the Mosquitoes *Aedes aegypti* and *Aedes albopictus* to Experimental Plant Infusions in Laboratory Bioassays. J. Chem. Ecol..

[14] Yang Y.Z., Wang Y., Li H., Zhuang J. (2019). Microbial diversity of Guangxi Liubao and Chongqing Bowl teas. Acta Tea Sin..

[15] Xin D.D., Li D.X., Zhang H. (2020). Chemical Changes of Different Kinds of Tea with the Processing. Food Res. Dev..

[16] Zheng P.C., Liu P.P., Wang S.P., Teng J.F., Lin Z.L., Gong Z.M. (2018). Comparative Analysis of the Aroma Components in Five Kinds of Dark Tea. Sci. Technol. Food Ind..

[17] Kind T., Wohlgemuth G., Lee D.Y., Lu Y., Palazoglu M., Shahbaz S., Fiehn O. (2009). FiehnLib: Mass Spectral and Retention Index Libraries for Metabolomics Based on Quadrupole and Time-of-Flight Gas Chromatography/Mass Spectrometry. Anal. Chem..

[18] Lindh J.M., Okal M.N., Herrera-Varela M., Borg-Karlson A.-K., Torto B., Lindsay S.W., Fillinger U. (2015). Discovery of an oviposition attractant for gravid malaria vectors of the *Anopheles gambiae* species complex. Malar. J..

[19] Singh H., Akhtar N., Gupta S.K. (2024). Biology of Mosquitoes. Mosquitoes: Biology, Pathogenicity and Management.

[20] Aguilar-Durán J.A., Hamer G.L., Reyes-Villanueva F., Fernández-Santos N.A., Uriegas-Camargo S., Rodríguez-Martínez L.M., Estrada-Franco J.G., Rodríguez-Pérez M.A. (2024). Effectiveness of mass trapping interventions using autocidal gravid ovitraps (AGO) for the control of the dengue vector, *Aedes* (*Stegomyia*) *aegypti*, in Northern Mexico. Parasites Vectors.

