# Supplementary material for: Liu-pao Tea as a Source of Botanical Oviposition Attractants for Aedes Mosquitoes
Source: Insects. 2025 Oct 17;16(10):1065. doi: 10.3390/insects16101065 (PMC12564205; doi:10.3390/insects16101065)
Supplement: Supplementary file 1 [file insects-16-01065-s001.zip › insects-3896424-supplementary.pdf]

**Table S1. Instrument parameters**

| Project | Parameters |
|---|---|
| Incubate Temperature | 60 °C |
| Preheat Time | 15 min |
| Incubate Time | 30 min |
| Desorption time | 4 min |
| Front Inlet Mode | Splitless Mode |
| Front Inlet Septum Purge Flow | 3 mL /min |
| Carrier Gas | Helium |
| Column | DB-Wax（30 m×250 μm×0.25 μm） |
| Column Flow | 1mL/min |
| Oven Temperature Ramp | 40 ° C hold on 4 min, raised to 245 °C at a rate of 5 °C /min, hold on 5 min |
| Front Injection Temperature | 250 °C |
| Transfer Line Temperature | 260 °C |
| Ion Source Temperature | 230 °C |
| Quad Temperature | 150 °C |
| Electron Energy | -70 eV |
| Mass Range | m/z:20-500 |
| Scan Mode | Scan |
| Solvent Delay | 0 min |
